# Minutes of Mississippi Valley Association of Dental Surgeons

**Published:** 1887-04

**Authors:** A. G. Rose, E. J. Betty


### Minutes of Mississippi Valley Association of Dental Surgeons.

FORTY THIRD ANNUAL MEETING, CINCINNATI, MARCH 2, 1887.

The Association was called to order by President Dr. E. G. Betty, with the Secretary, Dr. A. G. Rose, at his post.

Upon motion of the President, Dr. J. Taft offered prayer.

Upon motion, reading of the minutes of the previous session was dispensed with.

Dr. C. M. Wright, of the Executive Committee, reported the programme for discussion.

Dr. J. Taft moved that the sessions be from 9 A. M to 12 M.; 2 to 5:30 P. M. Carried.

Dr. J. Taft moved that 11 A. M. on Thursday be set apart as a special time for the election of officers. Carried.

Dr. Rose called the attention of the society to the death of Dr. E. Osmund, of Cincinnati, and J. P. Ullery, of Lawrenceburg, Ind., and moved that a committee be appointed to draft resolutions.

President Betty appointed Drs. J. Taft and A. Berry upon the committee.

Drs. Moore and Fletcher were appointed a Special Committee on Membership, and Dr. C. E. Miles, of Gallipolis, O., Dr. Grant Molyneaux, of Cincinnati, O., Dr. R. E. Wyatt, of Lockland, O., Dr. Levitt Custer, of Westerville, O., Dr. C. P. Gray, Madisonville, O., were elected members by the Secretary's ballot.

President Betty read an address on the history of the Mississippi Valley Association.

Dr. Betty's address was received and listened to with much interest and attention, and when closed Dr. Betty was greeted with well earned applause from all the members. The paper was exceedingly interesting to old and young members, and gave facts of interest in connection with the history of this, the oldest dental association in the world.

Dr. H. J. McKellops, of St. Louis, moved that a vote of thanks be extended to Dr. Betty for his admirable paper, and the motion being put by the Secretary, was carried unanimously.

Remarks of interest about this Association were made by Drs. A. Berry, J. Taft, H. J. McKellops, G. W. Keely, H. A. Smith, J. W. Jay, Jas. Leslie, D. W. Whaley, C. M. Wright, and others.

Upon motion, a committee consisting of Drs. E. G. Betty, J. Taft, and H. J. McKellops, were appointed to make an historical report of this Association.

Dr. J. R. Callahan, of Hillsboro, O., offered the following resolution :

WHEREAS, This Association having appointed a committee to prepare a history of this Society, recognize the fact that the collection of historical data necessary in the prosecution of the work, will entail sundry expenses upon said committee, and in view of which expense, be it

*Resolved*, That this Society pledges itself to reimburse said committee for such reasonable expenditures, and be it further

*Resolved*, That this Society agrees to remunerate said committee for time and labor expended in the prosecution of this important work.

FIRST DAY—AFTERNOON SESSION.

Meeting called to order by the President, and the minutes read and adopted.

Dr. Moore moved that the first subject be passed, and that the Society hear Dr. C. M. Wright's paper on "Nutrition," the second topic on the programme. The subject was discussed by Drs. Smith, Rawls, Sage, J. Taft, Berry, Wright, Fletcher, Harlan, McKellops, and Morrison.

SECOND DAY—MORNING SESSION.

Meeting called to order by President Betty, the Secretary at his desk.

Minutes of previous day were read and ordered adopted.

Dr. Jay moved that the third subject be taken up, that of a "Case in Practice," by G. W. Keely, who read an interesting paper upon "Irregularity Cases," showing diagrams and models to illustrate the different steps he had taken in the management of the cases. Dr. Keely, at the close of his excellent paper, was greeted with much applause.

Topic discussed by Drs. Jay, Wright, Berry, Keely, McKellops, Fletcher, and Morrison.

Vice President Dr. H. L. Moore took the Chair.

Dr. Sillitto moved that subject be passed, and that the Society hear a paper by Walter A. Dun, M.D., of Cincinnati, upon the relations of Medicine and Dentistry. Dr. Dun's paper was well received, and upon its conclusion Dr. Betty moved that a vote of thanks be extended to Dr. Dun for his excellent paper. Motion carried unanimously.

Dr. Dun then introduced a Mr. Folger, one of the best hypnotic subjects in Cincinnati, and after placing him under the influence of the mesmeric power, placed a pin through the skin of the right hand, the subject walking about the room showing his hand to the members. Subject felt no pain whatever. Subject was again placed under the power and caused to open his mouth, and to keep it open for some time. Subject stated he was unable to close his mouth, felt no pain at all while under the power, but a soreness of muscle when restored to consciousness.

Case reported and topic discussed by Drs. Smith, Fletcher, Keely, Wright, Stout, Harlan, Poore, and Rawls.

On motion, 2 o'clock was set apart as a special time for Dr. How.

SECOND DAY—AFTERNOON SESSION.

Meeting called to order by Vice President H. L. Moore.

Reading of the minutes was dispensed with.

Dr. How gave an exhibition of the workings of the Oxy-Hydrogen Blow Pipe and appliances for crown and bridge work with specimens of the work, and also exhibited "Impression Cups."

Dr. McKellops called the attention of the Association to the matrices of Ash & Sons, London, England; Dr. Miller, of Altoona, Pa., and Flannigan, of St. Louis, flannel disks for polishing, and the latter an improvement to the Bonwell mallet, showing specimens of each.

Dr. J. Taft exhibited an abscess syringe for injecting medicants into alveolar abscesses.

Mr. Liggett, of the Detroit Electric Motor Co., showed and explained their improved electric motor, adapted for dental engines; mallets and mouth lamp.

Dr. A. W. Harlan, of Chicago, read a paper upon "New Remedies," calling attention to the use of "Cocaine," "Iodol," "Aseptol," "Eulyptol," "Trichlor-Phenol."

Paper was received, and the topic discussed by Drs. Cassidy, Sage, Betty, Rawls, Wright, and Sillitto.

The exhibition of Dr. Patrick's appliances was made a special order of business for 9 o'clock on Friday morning, and Society adjourned until that time.

THIRD DAY—MORNING SESSION.

Meeting called to order by President Betty, the Secretary at his post.

Minutes of the previous day were read, and, after slight changes, were ordered adopted.

Dr. J. Taft, on behalf of the Committee on Resolutions on the death of Dr. Edmund Osmond, of Cincinnati, made the following report:

IN MEMORIUM.

Since the last meeting of this Society, one of its honored members, viz.: Dr. E. Osmond has been called away by death, and and it is eminently fit and proper that we should make record of the regard and respect in which he was held by his associates and confreres; therefore

*Resolved*, That in the death of Dr. Osmond this Society has lost one of its most constant and devoted members; for the last twenty years or more he was rarely if ever absent from the annual meeting of this body, and was always ready to take part in its proceedings.

*Resolved*, That not only does the Society sustain a great loss in the death of Dr. Osmond, but the profession has lost a faithful member, one who was justly entitled to the respect of all who knew him: he always evinced a great interest in all that pertained to his chosen profession, as a result of which, in several particulars, much, was by his skill and genius, added to its resources; its progress was ever a matter of solicitude to him; for his friends he had an unusually strong attachment, and in every way he was a person of strong and decided views.

*Resolved*, That we tender to the family our most sincere and heartfelt sympathy in this their great bereavement and sorrow.

*Resolved*, That a copy of these resolutions be sent in proper form to the family, and a copy furnished to the press for publication.

J. Taft,
A. Berry,
*Committee.*

Dr. A. Berry, on behalf of the committee appointed to draft resolutions on the death of Dr. J. P. Ullery, offered the following:

It having been the Lord's will to remove from among us Dr. J. P. Ullery, we record our tribute of respect to his memory. He was one of the founders of the Mississippi Valley Dental Association and a constant attendant upon its meetings.

In demeanor he was quiet and unobtrusive, industrious and careful in his operations, and possessed in a remarkable degree the respect and confidence of his patrons and of all who knew him.

By observance of strictly temperate habits, although not physically robust, he was able to meet the requirements of his profession, and finally passed away worn out by an unusually arduous practice during about fifty years.

To the bereaved members of Dr. J. P. Ullery's family, we tender our heartfelt sympathy, and direct that a copy of this resolution be spread upon the minutes of the Association, and a copy sent to the bereaved family.

A. Berry,
J. Taft,
*Committee.*

Tribute to their memory was paid by Drs. Leslie, Sage, Smith, Wright, J. Taft, Berry, G. W. Keely, and Morrison.

The special order of business was taken up, the Secretary read a letter written by Dr. J. J. R. Patrick, of Bellville, Ill., describing his Improved Regulator for moving natural teeth.

Dr. McKellops exhibited a gold crown, an improved porcelain crown, and an improved rubber dam holder, all made by Dr. Patrick.

The Treasurer, Dr. F. A. Hunter, made his annual report to the Association.

Balance $49.75. Report received.

The President appointed Drs. Wright and J. R. Callahan to audit accounts.

The Secretary reported bills for printing and postage to the amount of $10.75.

The Secretary was ordered to draw on the Treasurer for the amount.

Dr. Fletcher moved that an appropriation of fifteen dollars be made the Secretary and five dollars to the janitor. Carried.

The Association then proceeded to the election of officers for the ensuing year, with the following result:

President, Dr. A. W. Harlan, of Chicago.

First Vice-President, Dr. Wm. N. Morrison, of St. Louis.

Second Vice-President, Dr. M. H. Fletcher, of Cincinnati.

Corresponding Secretary, Dr. F. W. Sage, of Cincinnati.

Recording Secretary, Dr. A. G. Rose, of Cincinnati.

Treasurer, Dr. F. A. Hunter, of Cincinnati.

President Betty appointed Drs. Van Antwerp and McKellopps installation committee to conduct the newly elected President to the chair, Dr. McKellops making the introducing speech. Dr. Harlan responding in fitting language, thanked the Association for the honor conferred, and assumed the chair and gavel.

Dr. Wright moved that a vote of thanks be extended to the retiring officers for their efficient work. Carried unanimously.

Dr. Callahan, of the committee appointed to audit the accounts of the Treasurer, reported them correct. Upon motion the report was accepted and the committee discharged.

The question of the prize coming up, it was found by reference to last year's minutes that no one had complied with Dr. Betty's resolution. Question discussed by Drs. Harlan, Wright, Jay, Rose, Smith, Sage, Betty, Berry, Van Antwerp, Fletcher, and Leslie

Dr. Smith offered the following resolution:

*Resolved*, That a sum of twenty-five dollars be awarded to the person presenting the best essay or paper to the Chairman of the Executive Committee of this body one month before the annual meeting in March, 1888, said essay or paper to be upon a subject to be decided upon by the Executive Committee of this Association. Resolution carried.

President Harlan appointed the following committees:

EXECUTIVE.

C. M. Wright, H. J. KcKellops, and J. R. Callahan.

PUBLICATION.

E. G. Betty, W. H. Sillitto, and A. G. Rose.

MEMBERSHIP.

H. L. Moore, M. Stout, and A. O. Rawls.

ETHICS.

O. N. Heise, C. I. Keely, and Wm. Van Antwerp.

The appointment of the delegates to the American Dental Association referred to the Executive Committee.

Association then adjourned to meet the first Wednesday in March, 1888.

A. G. Rose,
*Recording Secretary.*
E. J. Betty,
*President.*

